# Industrial manufacturing dataset for cylindrical plunge grinding of martensitic gray cast iron piston rings

**DOI:** 10.1016/j.dib.2026.112706

**Published:** 2026-03-20

**Authors:** Mirelli de Castro Cesário, Marcos Vieira de Souza, Matheus Costa Pereira, Anderson Paulo de Paiva

**Affiliations:** Federal University of Itajubá (UNIFEI). Avenida BPS, 1303, Pinheirinho, Itajubá/MG 37500-903, Brazil

**Keywords:** Machining process data, Industrial manufacturing data, Response surface methodology, Process capability

## Abstract

This article presents a dataset generated from a cylindrical plunge grinding process conducted in an industrial piston ring manufacturing environment. The data aim to support studies focused on process capability, variability, robustness, parameter optimization, and modelling approaches in grinding research. The experimental conditions were defined following a Central Composite Design for *k* = 4 factors and axial distance ρ=1.5, which established the levels of Wheel infeed rate, Dressing speed, Grinding wheel peripheral speed, and Dressing depth. These parameter combinations were carried out on the shop floor under real production constraints, ensuring that the collected measurements represent industrial operating conditions rather than laboratory simulations. Two external noise factors were considered during data collection, consisting of the different mandrels and the position of the piston ring within the production package. These noise sources reflect variations commonly encountered in manufacturing and allow researchers to investigate the robustness and sensitivity of dimensional responses. A CCD with thirty runs, being sixteen factorial points, eight axial points, and six center points, organized in two blocks of experiments, was performed, associated with four noise combinations, defined according to an experimental plan. For every condition, ten repeated measurements, represented by ten piston rings sampled from the package and assembled in different mandrels, were acquired using the dimensional control fixture routinely employed in the industry, forming a dataset of 1200 runs. This procedure ensured consistency with existing quality inspection practices and provided a rich structure suitable for repeatability and uncertainty analyses. The dataset includes raw measurements, process parameters, and experimental identifiers that enable multilevel exploration of machining performance. Its structure supports diverse analytical applications, including modelling of process capability, variability, evaluation of noise effects, optimization of input parameters, statistical analysis of repeated measures, and the development or validation of data-driven and machine learning methods. Owing to its industrial origin, the dataset offers realistic variability patterns and is relevant for comparative studies, benchmarking activities, and the development of predictive or robust design frameworks in manufacturing research.

Specifications Table

**Table:** 

Subject	Engineering & Materials science
Specific subject area	Experimental data from an industrial plunge cylindrical grinding operation.
Type of data	Table
Data collection	The data were generated in an industrial environment during a plunge cylindrical grinding operation of martensitic grey cast iron piston rings. The levels of wheel infeed rate, dressing speed, grinding wheel peripheral speed, and dressing depth were initially defined using a Central Composite Design for *k* = 4 input factors and ρ=1.5. These parameter combinations were executed on the shop floor under real production conditions, which also included the controlled introduction of two noise factors: the chuck type and the ring position within the package. For each experimental condition, ten repeated dimensional measurements were collected using a dedicated fixture routinely employed in the manufacturing process.
Data source location	• Institution: Federal University of Itajubá; • City/Town/Region: Itajubá, Minas Gerais; • Country: Brazil.
Data accessibility	Repository name: GitHub – Tabular Dataset for Analysis of an Industrial Plunge Cylindrical Grinding Process Data identification number: https://doi.org/10.5281/zenodo.18602410 Direct URL to data: https://github.com/Matheuscp98/Grinding/
Related research article	None

## 1 Value of the Data

• Industry-based machining dataset: Provides data generated from a Response Surface design and was executed in an industrial piston ring manufacturing process, enabling structured exploration of the effects of Wheel infeed rate (x_1_), Dressing speed (x_2_), Grinding wheel peripheral speed (x_3_), and Dressing depth (x_4_);
• Controlled variability sources: Includes two noise factors (chuck type (z_1_) and ring position within the package (z_2_), allowing researchers to examine how external disturbances influence dimensional responses and to investigate robustness in grinding operations representative of actual shop-floor conditions;
• Rich within-condition structure: The dataset [1] follows a hierarchical experimental design that integrates controlled process conditions, external noise factors, and repeated measurements. A total of 30 experimental conditions were defined using a Central Composite Design. For each condition, four noise scenarios were evaluated, corresponding to combinations of mandrel configuration and ring position within the production package, with 10 replicated measurements collected per scenario. As a result, each experimental condition comprises 40 observations, totalling 1200 records;
• Multilevel applicability: In practical terms, the dataset supports a range of concrete research applications enabled by its structured design and industrial origin. For example, it can be used to model and quantify the influence of external noise factors on dimensional variability, supporting robustness analyses under realistic manufacturing disturbances. The repeated measurements further enable investigations of process variability and measurement repeatability, contributing to uncertainty quantification and industrial quality assessment. In addition, the dataset provides a structured benchmark for developing and comparing statistical or machine learning models aimed at predicting grinding performance or optimizing process parameters under controlled and noisy conditions;
• Machine learning applicability: The consistent design and repeated-measure structure make the dataset suitable for predictive modelling, feature selection, dimensionality reduction, variance decomposition, and validation of machine learning workflows applied to industrial manufacturing data.

## 2 Background

The increasing demand for tighter dimensional tolerances and high-quality surface finishes in components with complex geometries has driven advancements in manufacturing processes, particularly in the automotive and aerospace sectors. This demand reflects the pursuit of enhanced precision, performance, and durability in critical parts, thereby challenging conventional production methods and fostering the adoption of advanced technologies [2].

In recent decades, machining has been the most widely employed method for manufacturing products [3,4]. It is a manufacturing process used to provide the workpiece with its final shape, the desired surface properties, and the dimensional accuracy specified in the design [5]. Machining operations include turning, milling, grinding, drilling, and other processes [6,7], with widespread applications in automotive components, aerospace engines, and hydraulic systems [8].

Surface quality and productivity depend directly on the appropriate configuration of process parameters, which affect essential properties such as tool wear, tool life, surface finish, and material removal rate [9]. Studies that integrate mathematical modelling with quality and productivity measures further highlight the relevance of understanding parameter interactions in manufacturing processes [10]. The optimization of these parameters has a decisive impact on production efficiency, process quality, and operational costs [11,12].

**Fig. 1:**
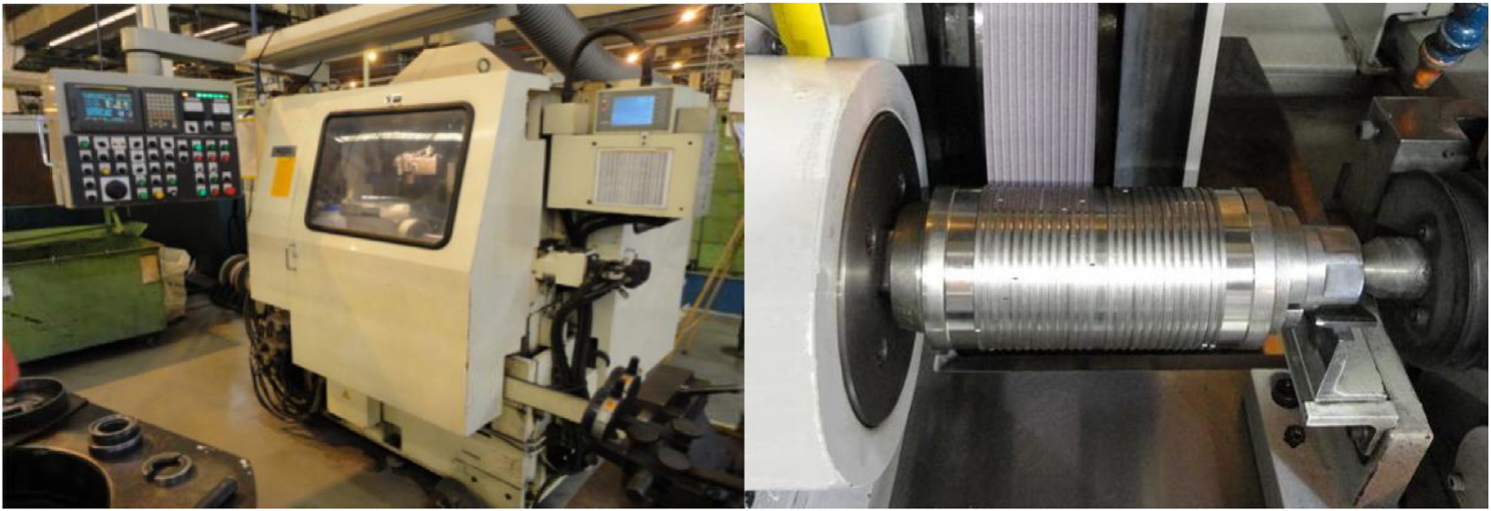
CNC cylindrical grinder.

The grinding process (Fig. 1) is influenced by numerous interacting parameters, as well as variability introduced by noise factors, which can compromise process consistency and product quality. Advanced optimization techniques based on multivariate criteria, such as principal component-based global criterion formulations, provide robust frameworks for evaluating multiple responses simultaneously [12]. To address these challenges, robust experimental methodologies are essential to avoid reliance on empirical trial-and-error approaches that are often costly and inefficient.

## 3 Data Description

**Fig. 2:**
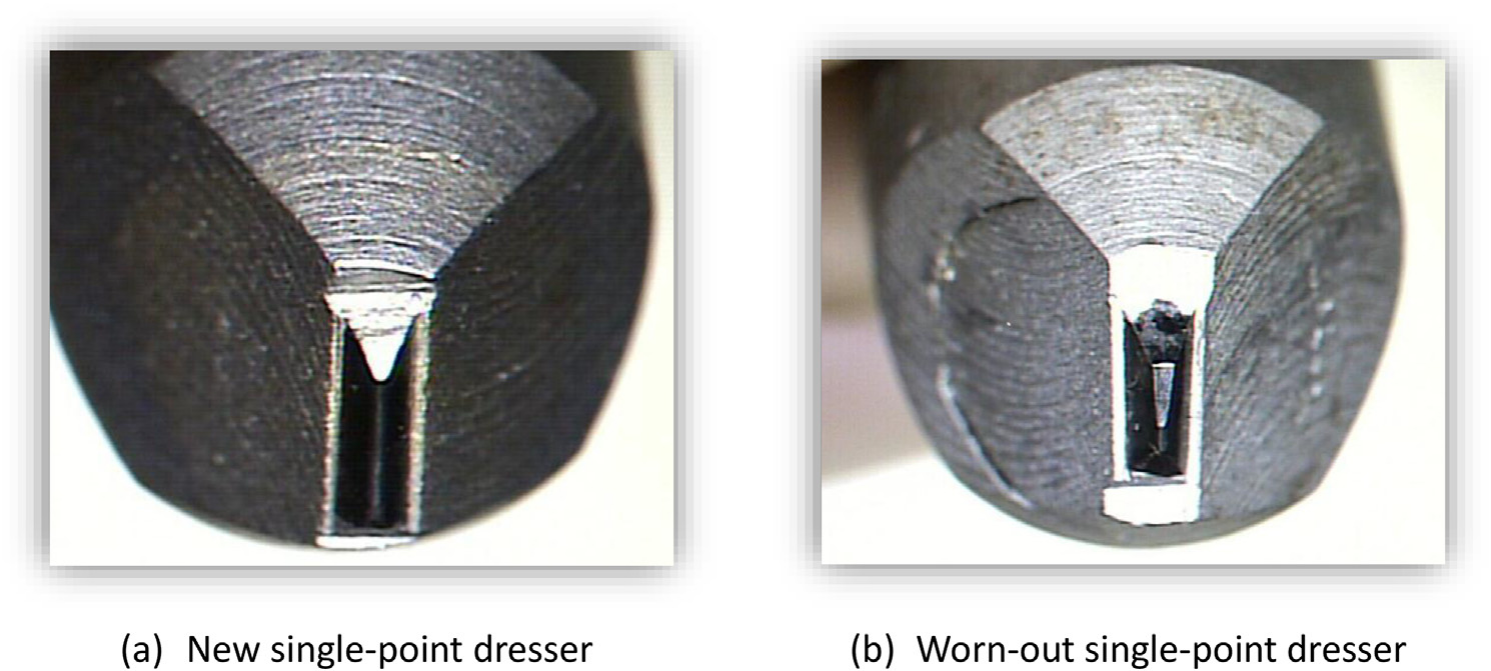
Single point dresser.

The experimental procedures were carried out using a CNC (Computer Numerical Control) machining for plunge cylindrical grinding operation, as shown in . The operation mode employed was plunge grinding, a common technique for achieving precise dimensional control in cylindrical components. For the dressing stage, a single-point diamond dresser equipped with a profiled natural diamond tip was utilized, as can be seen in Fig. 2.

Due to the wear of diamond dressers, the grinding forces may be affected during the grinding operation, causing severe fracture of the grains. For example, as a consequence, the surface roughness increases; besides that, the process variability also increases, which is exactly opposite to the requirements of tight tolerances. Moreover, vibration can occur during dressing, affecting the topography of the grinding wheel.

**Fig. 3:**
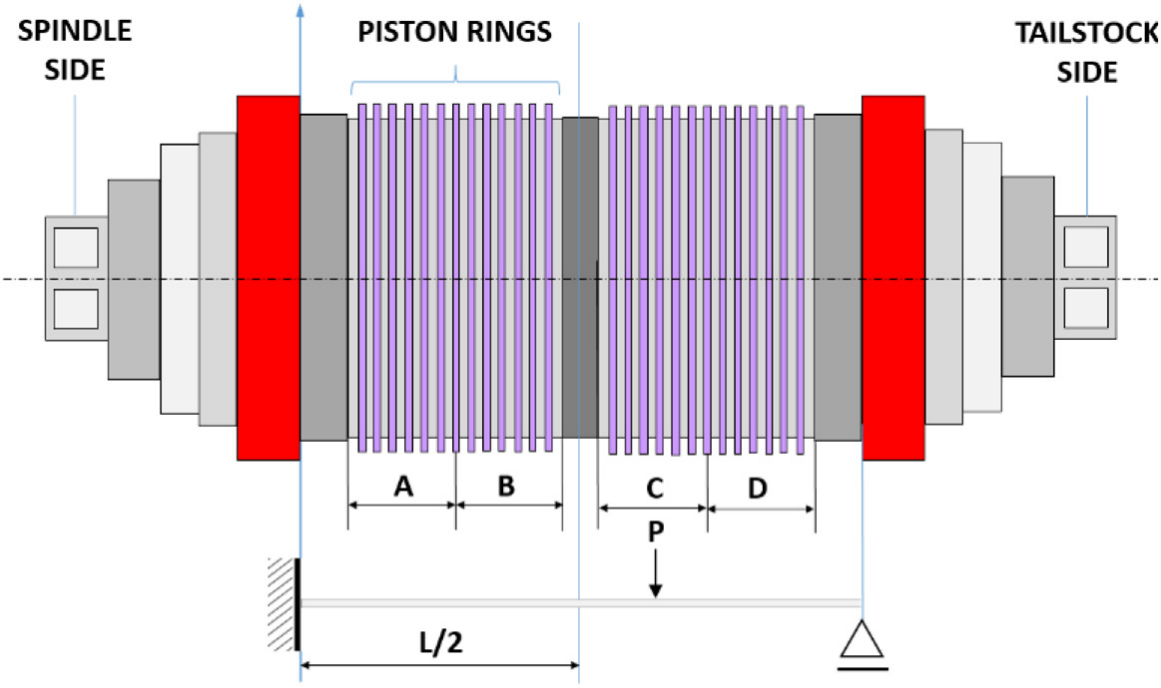
Schematic view of the assembly of piston rings on a mandrel.

The workpiece used in the experimental trials is a package of piston rings, which is electroplated on its outer diameter with a thin layer of hard chrome, approximately 0.10 mm thick, and with a minimum hardness of 800 Vickers hardness. The base material is a martensitic ductile cast iron. These piston rings (Fig. 3) feature a distinctive crowned profile defined by two radii, resulting in three critical dimensions that must conform to strict design specifications.

Table 1 summarizes the specification limits and target values associated with the three key dimensional responses, T_1_, T_2_, and T_3_. These parameters define the geometric tolerances to which the piston rings must conform.

**Table 1:** Piston Rings' outer diameter profile dimensions.

Dimension	T_1_ [µm]		T_2_ [µm]		T_3_ [µm]	
Specification Limits	LSL	USL	LSL	USL	LSL	USL
	1.00	7.00	30.00	60.00	10.00	22.00
**Target [mm]**	4.00		45.00		16.00	

**Fig. 4:**
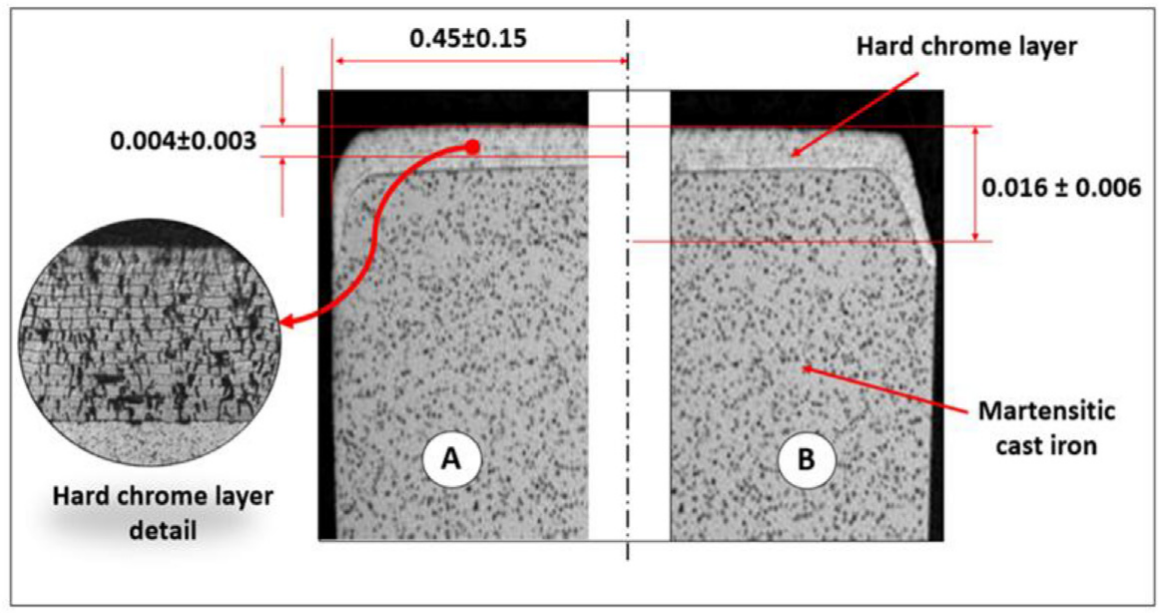
The cross-sectional profile and tolerances of a piston ring.

To support the interpretation of these dimensions, Fig. 4 provides a visual representation of the piston ring’s cross-sectional profile, highlighting the locations where T_1_, T_2_, and T_3_ are measured. This visual aid is essential for understanding the critical areas affected by the grinding process and their relevance to product quality.

The dataset and supplementary materials are publicly accessible in a GitHub repository [1], structured to support efficient reuse and facilitate transparent understanding of the variables involved. The repository provides clear documentation to enable applications in machine learning, statistical modelling, physical model development and validation, optimization studies, and academic training. Table 2 presents a comprehensive description of the dataset variables.

**Table 2:** Description of input and output variables.

Variable	Type	Abbreviation	Unit	Description
Wheel infeed rate	Input	x_1_	mm/min	Radial infeed rate applied during cylindrical plunge grinding, defined by the experimental design.
Dressing speed	Input	x_2_	mm/min	Relative speed between the dressing tool and the grinding wheel during dressing.
Grinding wheel peripheral speed	Input	x_3_	m/s	Tangential surface speed of the grinding wheel during the grinding operation.
Dressing depth	Input	x_4_	mm	Penetration depth of the dressing tool into the grinding wheel per dressing cycle.
Chuck type (Mandrel)	Input	z_1_	-	External noise factor representing the clamping configuration used during the grinding operation.
Ring position in the package	Input	z_2_	-	External noise factor indicating the position of the piston ring within the production package during processing.
Dimensional response	Output	T_1_, T_2_, and T_3_	µm	Piston Rings Output Dimensional measurements obtained for each experimental condition under different noise scenarios.

The dataset contains four input variables, consisting of Wheel infeed rate (x_1_), Dressing speed (x_2_), Grinding wheel peripheral speed (x_3_), Dressing depth (x_4_), and three output variables (T_1_, T_2_, and T_3_), which represent dimensional measurements obtained under industrial conditions. Two external noise factors are also recorded. The mandrel factor (z_1_) is categorized as mandrel 1 or mandrel 2, identifying the fixture used during machining. The ring position factor (z_2_) is categorized as A (left side) or B (right side) of the production package, indicating the physical location from which the ring was sampled. These categorical labels describe operational configurations rather than ordered levels and are intended to capture natural variability present in shop-floor conditions.

The dataset follows a hierarchical experimental structure that combines designed process conditions, external noise factors, and repeated measurements. A total of 30 experimental conditions were defined according to the Central Composite Design. For each condition, four noise scenarios were evaluated, corresponding to the combinations of mandrel configuration and ring position within the production package. Within each noise scenario, 10 replicated measurements were acquired. Consequently, each CCD condition comprises 40 observations, resulting in a total of 1200 records. This structure enables simultaneous analysis of controlled factors, noise-induced variability, and measurement repeatability.

The three-dimensional responses were generated simultaneously during the grinding process because the final ring profile is entirely determined by the geometry of the grinding wheel, which is configured through parameters programmed in the machine’s control panel.

Table 3 presents a summarized view of the dataset, where each row aggregates the dimensional responses associated with a given experimental condition. To explicitly illustrate how individual observations are structured, a representative data snippet is shown with records that share the same process parameters (x_1_ = 0.15 mm/min, x_2_ = 50 mm/min, x_3_ = 33 m/s, x_4_ = 0.02 mm) but differ in their noise configurations. For example, one observation corresponds to mandrel 1 and ring position A (left side), while another uses mandrel 2 with the same ring position, and a third corresponds to mandrel 1 with ring position B (right side). These variations demonstrate how the categorical noise factors z_1_ (mandrel 1 or 2) and z_2_ (A: left, B: right) are recorded alongside replicated dimensional responses (T_1_–T_3_), allowing the influence of operational variability to be examined within the same nominal machining condition.

**Table 3:** Representative subset of the central composite design (CCD) with summarized experimental results.

Replicate	x_1_	x_2_	x_3_	x_4_	z_1_	z_2_	T_1_	T_2_	T_3_
1	0.15	50.00	33.00	0.02	1	A	5.00	47.00	15.00
2	0.20	50.00	33.00	0.02	1	A	6.00	50.00	14.00
1	0.15	50.00	33.00	0.02	2	A	7.00	56.00	13.00
1	0.15	50.00	33.00	0.02	1	B	4.00	46.00	16.00

Table 4 presents a summarized view of the dataset, where each row aggregates the dimensional responses associated with a given experimental condition. In the first row, the data correspond to the experimental condition defined by a wheel infeed rate of 0.15 mm/min, dressing speed of 50 mm/min, grinding wheel peripheral speed of 33 m/s, and dressing depth of 0.02 mm. For this condition, T_1_, T_2_, and T_3_ represent the mean values computed from 40 measurements obtained across all noise scenarios.

**Table 4:** Central composite design (CCD) and a summary of the experimental results.

x_1_	x_2_	x_3_	x_4_	Dresser Life	Average T_1_	Average T_2_	Average T_3_
0.150	50	33	0.020	1207	4.475	48.050	15.550
0.200	50	33	0.020	1105	4.611	47.264	15.642
0.150	70	33	0.020	1090	5.100	47.450	16.100
0.200	70	33	0.020	1072	4.850	46.625	16.200
0.150	50	45	0.020	782	4.775	47.200	16.275
0.200	50	45	0.020	703	4.425	45.775	16.175
0.150	70	45	0.020	801	4.875	47.725	16.475
0.200	70	45	0.020	779	4.750	45.125	16.625
0.150	50	33	0.040	910	4.650	44.200	16.750
0.200	50	33	0.040	890	5.075	45.500	16.650
0.150	70	33	0.040	811	4.772	46.381	16.900
0.200	70	33	0.040	870	4.800	45.350	16.900
0.150	50	45	0.040	612	4.975	45.550	16.400
0.200	50	45	0.040	656	5.025	45.550	16.550
0.150	70	45	0.040	622	5.125	44.875	16.500
0.200	70	45	0.040	690	4.700	45.075	16.850
0.175	60	39	0.030	740	5.150	46.800	16.775
0.175	60	39	0.030	699	5.025	46.125	16.875
0.175	60	39	0.030	802	5.125	46.175	16.650
0.175	60	39	0.030	704	5.225	46.075	16.550
0.138	60	39	0.030	613	5.050	45.850	16.475
0.213	60	39	0.030	717	5.000	45.375	16.275
0.175	45	39	0.030	695	5.100	47.625	16.250
0.175	75	39	0.030	730	5.475	48.425	16.475
0.175	60	30	0.030	990	5.900	53.575	16.000
0.175	60	48	0.030	420	5.700	51.625	16.525
0.175	60	39	0.015	950	4.725	43.050	16.125
0.175	60	39	0.045	520	4.775	42.375	16.950
0.175	60	39	0.030	794	5.175	47.025	16.650
0.175	60	39	0.030	765	5.375	46.950	16.300

A subsequent row illustrates how different parameter settings lead to distinct aggregated responses. For example, the condition defined by a wheel infeed rate of 0.15 mm/min, dressing speed of 50 mm/min, grinding wheel peripheral speed of 33 m/s, and dressing depth of 0.04 mm is associated with a dresser life of 910 and mean dimensional responses of T1 = 4.650, T2 = 44.200, and T3 = 16.750, also calculated from the corresponding 40 measurements under all noise configurations. The complete dataset, including the individual records associated with each noise condition, is available in the repository for detailed inspection and reuse.

## 4 Experimental Design, Materials and Methods

The machine tool used in the experiments was a CNC cylindrical grinder, and the operation was the plunge cylindrical grinding. For dressing the grinding wheel was applied a single-point diamond dresser. The rings are fastened in a mandrel to be machined, forming two packs with 13 rings each, which are ground in two plunge steps. The first pack is on the left side, near the nose, and the second pack is on the right, near the tailstock. The grinding wheel applied in the experiments has the specification shown in Table 5.

**Table 5:** Grinding wheel characteristics.

Abrasive	Grit	Grade	Structure	Bond Type
RA	240	J	6	V16
Ruby Aluminum Oxide	Very Fine	Soft	Normal	Vitrified

Dimensional measurements were obtained using a Mahr MarSurf XC 20 contour measuring station. Calibration of this system is performed through an automated, software-assisted procedure (MarWin), based on tracing known reference profiles whose measured values are compared with certified standards. Calibration artefacts include gauge blocks, a standard thread profile, and a standard pin verified along the X and Z axes. An acceptance criterion of ±0.002 mm is adopted, and the calibration cycle is performed every 210 days. This procedure ensures measurement consistency and repeatability aligned with industrial inspection practices.

The first step of the methodology consisted of identifying and validating the noise factors that influence the dimensional profile of piston rings and, consequently, affect process capability. Two external noise factors were selected based on their expected contribution to process variability. The first was the positioning of the ring pack on the mandrel used to secure the rings during the grinding operation. Because each pack exhibits different stiffness levels, the pack positioned on the left side, near the spindle head, presents lower mechanical stability compared to the pack located closer to the tailstock. The second noise factor was the mandrel itself. Two mandrels assembled with slightly different components were used, and these small construction differences may introduce positioning errors during clamping, potentially altering the final profile dimensions.

**Fig. 5:**
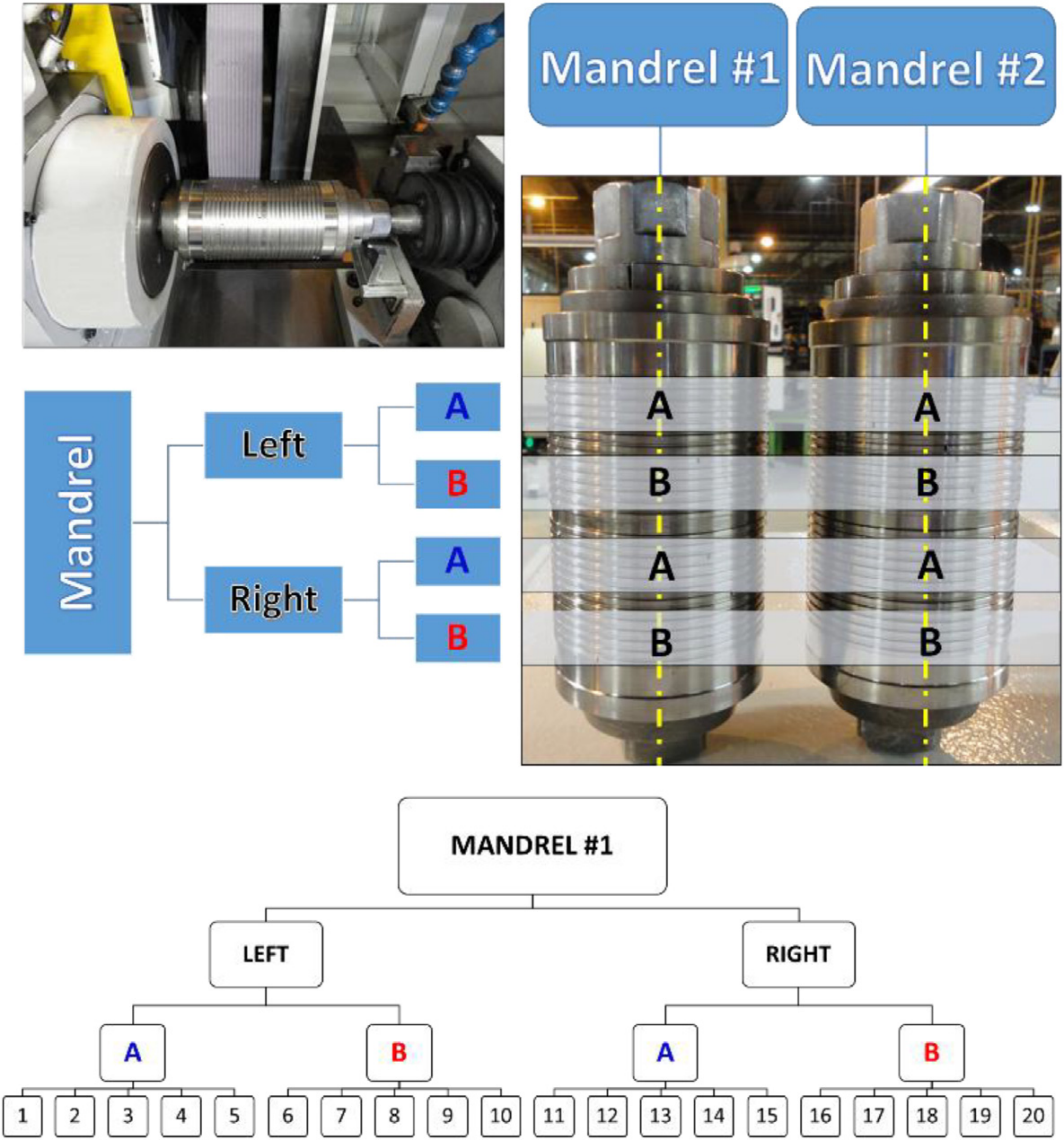
Illustration of the mandrel configurations and ring sampling positions used to define the external noise factors.

The noise factors are illustrated in Fig.5, which also helps clarify the hierarchical structure of the measurements. The figure represents how repeated measurements are organized for each combination of noise conditions. For illustration purposes, the hierarchical structure within mandrel 1 is shown, where it is possible to observe the ten measurements acquired for each ring position (left and right), highlighting how variability is captured within each operational configuration.

After defining the noise factors, the controllable process parameters selected for investigation in the Response Surface Methodology design included the wheel infeed rate, the dressing speed, the grinding wheel peripheral speed, and the dressing depth. The specific parameter levels for these variables are presented in Table 6. These controllable factors were chosen based on prior process knowledge, practical feasibility, and their anticipated influence on the dimensional responses.

**Table 6:** Levels of the controllable factors.

Process Parameters	Unit	Levels				
		−1.5	−1	0	+1	+1.5
x_1_ - Plunge infeed rate	[m/min]	0.137	0.150	0.175	0.200	0.212
x_2_ - Dressing speed	[m/min]	45.000	50.000	60.000	70.000	75.000
x_3_ - G.W. surface speed	[m/s]	39.000	33.000	39.000	45.000	48.000
x_4_ - Dressing depth	[mm]	0.015	0.020	0.030	0.040	0.045

The experimental matrix was constructed using a Central Composite Design. Four controllable factors were tested at two coded levels (2⁴ = 16), supplemented by six center points (1 × 6 = 6) and eight axial points (2 × 4 = 8). A single replication was executed, and the axial distance (ρ) was set to 1.50, resulting in a total of 30 experimental runs. Within each experimental block, the execution order of the CCD runs was randomized to minimize potential bias associated with temporal effects, machine drift, or operational variability.

Several process conditions were held constant throughout the experiments, including a stock removal of 0.12 mm on the diameter, a grinding wheel of type RT 510 × 52 × 203.2 – DR 240 J6 V16, and a workpiece rotation speed of 150 rpm. The coolant applied was Ecocool 1977, delivered at a flow rate of 20 liters per minute.

## Limitations

The dataset presents a number of limitations that should be acknowledged when interpreting or reusing the data. Although the experiments were conducted in an industrial environment at a multinational piston ring manufacturer, the machining conditions reflect the specific operational constraints of that facility, which may limit generalization to other tooling systems or machine setups.

The parameter levels for Wheel infeed rate, Dressing speed, Grinding wheel peripheral speed, and Dressing depth were defined using a Response Surface Methodology design, restricting the dataset to a predefined experimental region. Extrapolation beyond these ranges is therefore not supported. Additionally, only two external noise factors (chuck type and ring position within the package) were introduced. While these factors improve robustness by introducing realistic variability, they do not represent the full spectrum of disturbances found in production environments.

Although the dataset includes 30 experiments, four noise combinations, and ten repeated measurements per condition, the sample size may be modest for high-complexity modelling tasks. Furthermore, dimensional measurements were obtained indirectly through a dedicated fixture rather than by direct metrology. This standard industrial practice is adequate for process control, but it may introduce uncertainties related to fixture alignment and repeatability.

## Ethics Statement

The authors have read and follow the ethical requirements for publication in Data in Brief and confirm that the current work does not involve human subjects, animal experiments, or any data collected from social media platforms.

## CRediT Author Statement

**Mirelli de Castro Cesário:** Conceptualization, Methodology, Writing – original draft**; Marcos Vieira de Souza:** Data curation, Writing – original draft; **Matheus Costa Pereira:** Conceptualization, Writing – original draft; **Anderson Paulo de Paiva:** Supervision, Writing – review & editing**.**

## Data Availability

GithubTabular Dataset for Analysis of an Industrial Plunge Cylindrical Grinding Process (Original data).
